# A new paradigm in respiratory hygiene: modulating respiratory secretions to contain cough bioaerosol without affecting mucus clearance

**DOI:** 10.1186/1471-2466-7-11

**Published:** 2007-08-13

**Authors:** Gustavo Zayas, Juan C Valle, Mauricio Alonso, Henry Alfaro, Daniel Vega, Gloria Bonilla, Miguel Reyes, Malcolm King

**Affiliations:** 1 University of Alberta Pulmonary Research Group, Edmonton, Canada; 2 Hospital Nacional "Dr. Jose Antonio Saldaña" Neumologia y Medicina Familiar, San Salvador, El Salvador

## Abstract

**Background:**

Several strategies and devices have been designed to protect health care providers from acquiring transmissible respiratory diseases while providing care. In modulating the physical characteristics of the respiratory secretions to minimize the aerosolization that facilitates transmission of airborne diseases, a fundamental premise is that the prototype drugs have no adverse effect on the first line of respiratory defense, clearance of mucus by ciliary action.

**Methods:**

To assess and demonstrate the primary mechanism of our mucomodulators (XLs), we have built our evidence moving from basic laboratory studies to an *ex-vivo *model and then to an *in-vivo *large animal model. We exposed anesthetized dogs without hypersecretion to different dose concentrations of aerosolized XL "B", XL "D" and XL "S". We assessed: cardio-respiratory pattern, tracheal mucus clearance, airway patency, and mucus viscoelastic changes.

**Results:**

Exposure of frog palate mucus to XLs did not affect the clearance of mucus by ciliary action. Dogs maintained normal cardio-respiratory pattern with XL administration. Tracheal mucociliary clearance in anesthetized dogs indicated a sustained 40% mean increase. Tracheal mucus showed increased filance, and there was no mucus retention in the airways.

**Conclusion:**

The *ex-vivo *frog palate and the *in-vivo *mammalian models used in this study, appear to be appropriate and complement each other to better assess the effects that our mucomodulators exert on the mucociliary clearance defence mechanism. The physiological function of the mucociliary apparatus was not negatively affected in any of the two epithelial models. Airway mucus crosslinked by mucomodulators is better cleared from an intact airway and normally functioning respiratory system, either due to enhanced interaction with cilia or airflow-dependent mechanisms. Data obtained in this study allow us to assure that we have complied with the fundamental requirement criteria established in the initial phase of developing the concept of mucomodulation: Can we modulate the physical characteristics of the respiratory secretions to reduce aerosolization without affecting normal mucociliary clearance function, or even better improving it?

## Background

The respiratory system performs one of the most vital functions of the body: the respiratory process, that consists of an appropriate gas exchange occurring in the lungs between blood and air. It provides oxygen to the organism and removes carbon dioxide. Air has to travel through a series of passages, and interact with several types of tissues, until it reaches the alveolus where the gas exchange takes place. Air is in contact with many bacteria, pollutants and particles in the environment before reaching the alveolus. Many of these components are inert participants, but others are very harmful, and can disable the airways natural defence mechanisms against injuries. Hence, air must be cleaned, warmed and humidified.

There are several mechanisms to protect such a vital function. Two main complementary mechanisms act to maintain the airways as free as possible of foreign bodies and the airway epithelium from damage due to inhaled pathogens and respiratory insults: namely the mucociliary clearance and the cough clearance. The former constitutes the first line of defence and the latter is the backup or reinforcement if the first one is unable to carry out the task.

Mucus is a unique *materia *lining all the major body cavities, produced by the same type of cells: the goblet cells and the mucous cells of the submucosal glands. In the lungs, other types of cells produce materials that contribute to the unique physical properties of mucus, like the Clara cell, and the type II pneumocyte, which produce the surfactant factor [1,2]. Mucus can be considered a viscoelastic fluid, since it exhibits both liquid-like (viscous) and solid-like (elastic) properties [3]. The relative proportions of elasticity and viscosity are as important in describing how a material such as mucus behaves when it is subjected to external forces, as are the absolute values of either parameter by itself.

The mucus lining the airways is a mobile layer constantly cleared by intimate interaction with cilia. It is also possible that interaction of mucus with the low velocity airflow during normal breathing might play a role in mucus clearability. This concept has not been well studied yet. Any change occurring in one component of the mucus-cilia unit might lead to disruption of normal mucociliary clearance processes, reducing lung defences and allowing respiratory secretions to accumulate and impair pulmonary function [4].

We are using an integrated approach to gain more insight into what might occur when the airway mucus-cilia unit is exposed to novel agents that selectively raise elasticity relative to viscosity of one of the components: the mucus macromolecule. This is the reason why we are also reviewing some theoretical aspects of these components.

The mucus macromolecule (mucin) has a basic, common and complex biochemical structure. Mucin macromolecules consist of oligosaccharide units attached to a proteic core [5,6]. The physical characteristics of mucus depend principally on the type and amount of glycoproteins. Viscosity and elasticity are the physical characteristics considered essential for mucus to function [7], although adhesivity and spinnability are particularly important in describing the cough clearability of airway mucus [8].

The frequency of mechanical response is important for mucus clearance, particularly in the airways. There is an optimum range among the physical properties that give mucus its capacity to behave in a balanced manner even at very different frequencies or rates of displacements. Low frequency or low amplitude is related to bicellular clearance, while high frequency/high amplitude is more closely related to cough clearance [8].

It should be noted that the viscoelasticity relevant to cough clearance is not necessarily the same as the viscoelasticity relevant to mucociliary clearance. Mucociliary clearance is defined by low frequency, low amplitude conditions, while cough clearance is governed by high frequency, high amplitude conditions. While there is generally an association between the low and high frequency/amplitude forms of viscoelasticity, it is possible to dissociate these parameters to some degree.

Respiratory infection alters mucus, which becomes thickened with the byproducts of the infection and requires appropriate treatment and possibly physiotherapy and agents that disrupt the intermolecular interactions of the airway mucus. This approach has clearly improved the survival rate of infected patients. Yet altering the physical properties of mucus while not respecting the effects on mucus barrier function and aerosolizability of expectorated secretions could lead to problems for both the patient and the surrounding community.

We implemented this study to test the effects that a specialized new generation of compounds might have on the clearance of mucus by ciliary action. This innovative therapy aims to modulate the mucin gel viscoelasticity by increasing the concentration of crosslinking sites in the mucin glycoprotein gel network, and/or forming poorly soluble mucin complexes towards what might be described as making the mucus "thicker" (a Respiratory Medicine heresy?), more cohesive, more resistant to breakup during high speed airflow interaction.

In this study we are starting to build the evidence that the proposed agents have no adverse effect on the first line of respiratory defence, namely clearance of mucus by ciliary action hence assess a previously established fundamental criterion: Can we modulate the physical characteristics of the respiratory secretions to reduce aerosolization without affecting normal mucociliary clearance function, or even better improving it? [9].

## Methods

To assess the effect of the mucomodulators on the clearability of airway mucus by ciliary action on a ciliated epithelium and on the intact airways we moved from basic laboratory to our *ex-vivo *frog palate exposure model then to an *in-vivo *large mammal model. When working with large mammals we also assessed the effect of the mucomodulators on the cardio-respiratory pattern, tracheal mucus clearance, airway patency and mucus viscoelastic changes. In this study we report the results on mucus clearance by ciliary action after exposure to mucomodulators.

### Exposure models

#### Frog palate preparation

From a bullfrog, *Rana catesbiana*, the upper portion of the head was removed following the procedures described in previous work [10,11], by cutting with scissors through from the junction of the posterior pharynx and esophagus out to the skin of the back and double pithing. This procedure was carried out after lowering the body temperature of the frog for 30 – 60 minutes inside a refrigerator to abolish pain sensations. The palate was examined for macroscopic lesions, such as ulcers, petechia or redness as evidence of inflammation. Only palates free of inflammatory indicators were included in this study. Any blood remaining in the epithelial surface was carefully washed away, then the excised head was placed palate side facing upwards on a piece of gauze saturated with frog Ringer solution (FRS) in a Petri dish.

The palate was placed inside the frog chamber, a wooden box with a glass top and fitted glass front, and manipulated through glove openings. The humidity inside the box was maintained near 100% using a Pari jet nebulizer, and the temperature was kept between 22° and 24°C by a rheostat-controlled, externally mounted light source. Before carrying out any measurement, the palate was allowed to stabilize inside the box for 15 minutes.

The FRS was prepared by mixing standard Ringer injection with sterile water (2:1). The composition of standard frog Ringer (in mmol/l) is 90 NaCl, 3 KCl, 2 CaCl2, and 15 NaHCO3 (220 mosm/l). The experimental procedures involving animals were approved by the Health Sciences Animal Policy and Welfare Committee, University of Alberta

#### Mucus transport velocity (MTV) determination in the frog palate

The palate was placed under a dissecting stereomicroscope provided with a reticulated eyepiece. Mucociliary clearance was determined by observing the movement of particles of charcoal powder gently deposited on a sample of frog mucus on the palate surface; its clearance was visually monitored and MTV determined. The displacement of 3 – 5 μL of endogenous frog mucus sample was calculated by dividing the distance traveled by the transit time across the 0.3 in (7.62 mm) segment marked between 0.1 and 0.4 inches in the graduated eyepiece. To determine the mucus transport velocities of the control and of the exposed, at least five measurements of the time required for the mucus sample to travel the defined distance were made.

#### Mammalian exposure model

Random source, mixed-breed dogs of either sex, free of respiratory tract infections or other observable respiratory tract pathology, weighing from 15 to 20 kg, were employed in this study. Fasting experimental animals were pre-medicated with xylazine (2 mg/kg i.1 m.), and then a vein in either anterior leg was catheterized. After observing a good flow rate of the solution administered intravenously, the dog was anesthetized with the lowest dose of sodium pentobarbital (15 – 20 mg/kg i.v. slowly administered) required to allow the animal to breathe spontaneously and to suppress ocular reflexes. Supplemental doses of sodium pentobarbital in bolus were administered as needed during the experiment.

The dogs were placed in supine position, immediately intubated as shallowly as possible with a # 8 to 9 endotracheal tube, and covered to minimize the fall in core temperature. Several manoeuvres were implemented to make sure that the ETT was inside the trachea, and only then was its balloon inflated and the tube fixed with tape to the upper jaw and to the fangs to avoid displacement.

#### Vital signs

heart rate, breaths per minute, temperature, and respiratory-cardiac pattern were constantly monitored throughout the experiment. During the experiment each animal inhaled either nebulized normal saline solution (sham exposure) or nebulized mucomodulators diluted in normal saline solution (0.9% NaCl) in three different concentrations. They were slightly hyperosmolar compared to normal saline solution, except for the highest dose of XL "B" (sodium tetraborate, 0.1 M), which reached an estimated osmolarity of 0.6 Osm, the equivalent of *ca*. 1.8% NaCl. XL "D" (high molecular weight dextran), based on a macromolecule, increases osmolarity very minimally. After the experiment the animals were euthanized with an overdose of sodium pentobarbital plus potassium chloride intravenously and the trachea was excised for histopathological studies.

#### *In-vivo *tracheal mucus velocity (TMV) in dogs

Direct evaluation of mucus clearability was determined by a bronchoscopic technique. A drop (10 μl) of a suspension of powdered charcoal in saline solution was gently deposited onto the right side of the lower tracheal wall, approximately 1 cm above the carina. The position of the moving leading edge of the charcoal spot was located by advancing the tip of the bronchoscope to lay above the spot.

The exterior end part of the endotracheal tube allowed us to mark that position in the bronchoscope as the initial point. Setting the chronometer for five minutes, we started retracting the bronchoscope as the leading edge of the charcoal spot approached the upper part of the trachea, then marking on the bronchoscope the final point where the spot reached the internal tip of the ETT, or at the end of the five minutes period, and then measuring the distance between those two points. Transit times were five minutes or less depending on the rapidity with which the charcoal front approached the inlet of the endotracheal tube. TMV was computed as the distance in millimeters traveled divided by the time elapsed in minutes.

TMV measurements were made: a) once immediately after initial intubation of the trachea, prior to any drug intervention (baseline), b) twice after 30 minutes aerosolization of saline solution (control), and c) twice after 20 minutes of each dose of the experimental drug delivered by aerosol (six measurements). Three aerosol concentrations were delivered: Low = 0.001 M; Med = 0.01 M; High = 0.1 M. We made a total of nine mucociliary clearance measurements per animal per experiment.

The first sample of mucus (baseline) was obtained using the brush technique and no removal of the ETT was required. Then the ETT was removed immediately after every TMV measurement to allow for mucus collection, and rapidly a second endotracheal tube was introduced and fixed after both doses of saline and after both doses of the low, medium and high concentration delivery of test drugs. Every dog had the ETT removed and reinserted eight times during the experiment. After every tracheal intubation the animal was allowed to rest during five minutes while normal saline solution was delivered by aerosol.

### Mucus collection

#### Brush technique

The brush technique was used only once at the beginning of the experiment for baseline determination. Mucus was collected using the bronchoscopic technique developed by Jeanneret-Grosjean *et al *[12] from the same tracheal level but in the opposite side from where charcoal was deposited for TMV measurement. A cytology brush was gently placed in direct contact with the tracheal mucosa for five minutes, thus allowing for the collection of several microliters of mucus. The mucus sample was scraped from the brush and immediately covered with light paraffin oil and stored in a freezer at -30°C.

#### Endotracheal tube technique

Airway mucus was collected: a) twice after aerosolization of saline solution (as control), and b) twice after aerosolization of the experimental drug in low (0.001 M), medium (0.01 M) and high (0.1 M) concentrations (six collections) using the endotracheal tube (ETT) technique [13] for a total of eight ETT mucus sample collections per animal.

Immediately after every aerosolization and TMV measurement, the dog was extubated to collect mucus adhering to the tube. Another clean endotracheal tube was immediately placed in the trachea. The mucus coating the tube was covered with light paraffin oil (No. 0-121; Fisher Scientific, Fair Lawn, NJ), to prevent dehydration and removed by scraping the ETT with the edge of a forceps, placed into a 15 ml plastic test tube containing about 8 ml of light paraffin oil and stored in a deep freezer under -30°C. This method optimizes the quantity of mucus collected, which is important for the development of further biochemical assays.

### Mucomodulator exposures

#### Frog palate exposure

We prepared several dilutions of the mucomodulators employed (from 10^-1 ^M to 10^-8 ^M). We determined the baseline MTV after 15 min of stabilization of the palate inside the frog box, previous to any intervention. We exposed the palate by gently depositing 5 μl of the dilution of the agent to test on the proximal portion of the palate, proceeding from the lowest concentration to the next high concentration.

After baseline determination, we followed a pattern of 20 minutes period of rest under 100% humidity and room temperature inside the frog box. Then timing every run to measure MTV five times after applying through an Eppendorf micropipette a) 5 μl of frog Ringer (control), b) 5 μl of XL 10^-8 ^M, c) 5 μl of XL 10^-7 ^M, let the palate rest for 20 minutes, then MTV measure again after d) 5 μl of frog Ringer, e) 5 μl of XL 10^-6 ^M, f) 5 μl of XL 10^-5^, let the palate rest for 20 minutes, and so on until concentration 10^-1 ^M. At the end of the experiment we have performed one MTV baseline measurement, three MTV after FR (control) and eight MTV after every different concentration of XL.

#### *In-vivo *dog exposure

We exposed spontaneously breathing, lightly anesthetized dogs to three different concentrations of both mucomodulators XL "B" and XL "D" (10^-3 ^M, 10^-2 ^M and 10^-1 ^M) and to normal saline solution XL "S" as sham exposure. Drugs were delivered by aerosol through an endotracheal tube using a Pari LC Star jet nebulizer system set at 8 l/min measured by a Puritan flow meter. We nebulized 5 – 7 ml of each concentration of both mucomodulator XL"B" and XL"D" for about 20 minutes (~0.35 ml/min) for a dose of approximately 0.1 mg/kg, 1 mg/kg and 10 mg/kg per mucomodulator.

#### Baseline TMV

We measured the tracheal mucus velocity baseline, as explained above, before any intervention was performed. This was followed by collection of a mucus sample using the brush technique.

#### Control measurements

Normal saline solution was delivered by aerosol during 30 minutes, and then we measured TMV (a) for five minutes. After the clearance of mucus was measured the endotracheal tube was removed for mucus collection (i). Another endotracheal tube was immediately inserted inside the trachea and fixed, and saline solution was aerosolized for five more minutes; after this period the animal was left to breathe spontaneously for another five minutes.

After the period of rest, TMV (b) was measured again for five minutes; we then removed the ETT for mucus collection (ii) and another tube was reinserted, followed by a period of aerosolized saline solution and a period of rest, both for five minutes.

#### Mucomodulator exposures

The mucomodulator to test was randomly selected prior to initiating the experiment. A solution in low concentration (0.001 M) of the selected mucomodulator (XL "S", XL "B" or XL "D") was delivered by aerosol during 20 minutes, and then we measured TMV (c) for five minutes. After the clearance of mucus was measured the endotracheal tube was removed for mucus collection (iii). Another endotracheal tube was immediately inserted inside the trachea and fixed, and saline solution was aerosolized for five more minutes, after this period the animal was left to breathe spontaneously without any intervention for another five minutes.

Immediately after the rest period, TMV (d) was measured again for five minutes, and then the ETT was removed for mucus collection (iv) and another tube was inserted and fixed, followed by periods of aerosol of saline solution and of rest, both for five minutes.

The same procedure described above for XL low concentration was used for delivering the subsequent XL medium (0.01 M) and XL high (0.1 M) concentration solutions of the selected mucomodulator.

#### Tracheal tissue samples

Following the second TMV measurement after the administration of the high dose of XL and removal of the ETT, an overdose of a cocktail of sodium pentobarbital plus potassium chloride was administered intravenously in bolus to the animal. Through an open chest incision the trachea and both mainstem bronchi were excised, and immersed in formalin 20% for immunology and histopathology studies.

#### Mucomodulator agents

We have six different approaches that have the potential to accomplish these goals that we intend to test and develop [9]. However, in this study we report the effect of two of them, XL "B" and XL "D" as well as XL "S" (normal saline solution) the sham exposure, on the frog palate and on the respiratory airway of lightly anesthetized dogs.

One approach we tried was the use of sodium tetraborate solutions (mucomodulator "B", XL "B"), which cause reversible crosslink formation between galactose units that are the major neutral sugar component of mucins. In model studies using vegetable polysaccharides, sodium tetraborate preferentially raises elasticity relative to viscosity, and would favor mucociliary clearability at the expense of cough clearability and aerosolizability [3]. We have recently reported studies on the effect of added borate on the clearability and aerosolizability of mucus simulants. The results were quite striking: we were able to achieve increased "expectoration" in our cough machine model, along with normal mucociliary clearance in the frog palate model, and yet achieve our desired target of a significant reduction in fine aerosol formation [9].

A second approach was to administer high molecular weight dextran (mucomodulator "D", XL "D). HMW dextran has approximately the same molecular weight as the subunits of mucin macromolecules; in this case mucin-dextran crosslinks are approximately as effective as the original mucin-mucin crosslinks. Interestingly, HMW dextran tends to raise elasticity relative to viscosity (as indicated by the increase in spinnability); thus its use would tend to inhibit aerosolizability, which will depend on spinnability [14] while maintaining mucociliary clearability.

Dextran is a natural product used in the food, biochemical and pharmaceutical industries for more than five decades. *In vitro *synthesis of microbial dextrans is obtained from the action of dextransucrase from *Leuconostoc mesenteroides *on a sucrose substrate. Dextran molecular weight and the degree of branching can be influenced during synthesis by variables like substrate concentration and temperature.

### Statistical analysis

Data are expressed as mean ± standard deviation unless otherwise stated. A paired Student-T test was used for simple comparison. Newman-Keuls analysis was applied for repeated measurements. The Bonferroni correction was also used for statistical adjustment. The level of significance was set at 5%.

## Results

All frog palates used in these experiments appeared macroscopically healthy, and after the stabilization period inside the frog box, all showed normal ciliary clearance function.

### Epithelial exposure

The frog palate exposed to XL "B" had a baseline measurement of mucus cleared by cilia of 0.36 mm/sec and showed an average of MTV control measurements of 0.36 mm/sec. Applying the lowest concentration of the mucomodulator XL "B" (10^-8 ^M), immediately after the determination of the duplicate control measurement, it showed an average of 0.43 mm/sec in both sets of runs. Following with the next concentration (10^-7 ^M) we obtained an average of 0.45 mm/sec in both sets of runs.

**Figure 1:**
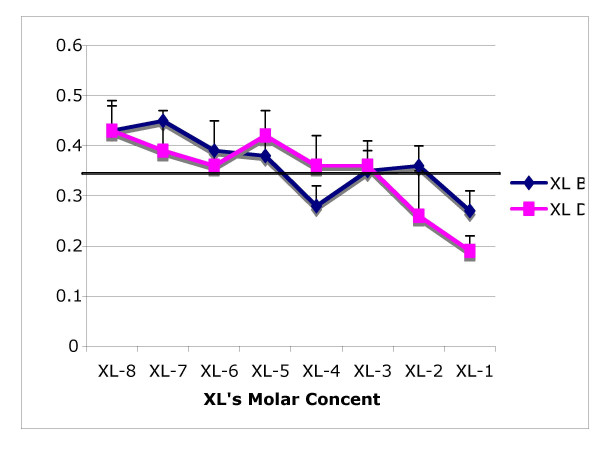
Effect on mucus transport velocity of the frog palate after topical application of eight different molar concentration of mucomodulators "B" and "D". Duplicate (five runs) measurement per control (four) and per dose (eight) plus one baseline measurement. (Total runs per experiment: 65). Control Grand Mean: 36 mm/min.

After testing all the four control measurements and the eight different concentrations we obtained a grand average of 0.37 mm/sec for the controls and 0.36 in mucus clearance velocity for all the XL "B" exposure. Comparison of both groups, control and experimental, using the *t*-test showed no statistical difference. However we noticed a decreasing trend in the transport rate as time elapsed (Figure 1).

The frog palate exposed to XL "D" had a baseline measurement of mucus cleared by cilia of 0.35 mm/sec and showed an average of MTV control measurements of 0.36 mm/sec. Applying the lowest concentration of the mucomodulator XL "D" (10^-8 ^M), immediately after the determination of the duplicate control measurement, it showed an average of 0.43 mm/sec. Following with the next concentration (10^-7 ^M) we obtained an average of 0.39 mm/sec in both sets of runs.

After testing all the four control measurements and the eight different concentrations we obtained a grand average of 0.36 mm/min for the control and 0.35 mm/sec in mucus velocity clearance for the XL "D" exposure. Comparison of both groups, control and experimental, using the *t*-test showed no statistical difference.

### Mammalian exposure

A total of seven male and six female dogs, 16 kg weight average, were exposed to mucomodulators: six of them to XL "D" (4 M – 2 F), five to XL "B" (2 M – 3 F) and two to XL "S"(1 M – 1 F). The skin temperature of the dogs remained stable around 34°C throughout the experimental time. The respiratory and cardiac frequency as well as the respiratory pattern remained with normal rhythm also stable at rate (12 breaths/min, 60 beats/min), except when the animal was restless when awakening and required more anesthetic.

All of the animals involved had at the start of the experiment a normal healthy looking airway mucosa, with no hypersecretion, no ulcers and no visible inflammation. Of all experimental animals used, five dogs yielded either a very small sample of mucus (4) or no sample at all (1) when using the baseline brush technique collection. One dog that yielded a very small sample using the brush technique consistently yielded either a small sample or no sample at all throughout the experiment while collecting mucus using the ETT mucus collection technique.

During the mucus collection using the ETT technique it was observed that in dogs that received either XL "D" or XL "B", even when using the lowest concentration, but not in those sham exposed (XL "S"), the mucus samples were mostly collected from inside the tube and very little from the outside portion of the tube. It was also observed that the samples of mucus collected from the inside or outside portion of the ETT in dogs that inhaled mucomodulators slid off the tube very easily when it was placed in a vertical position. The latter was not observed in those dogs sham exposed.

**Figure 2:**
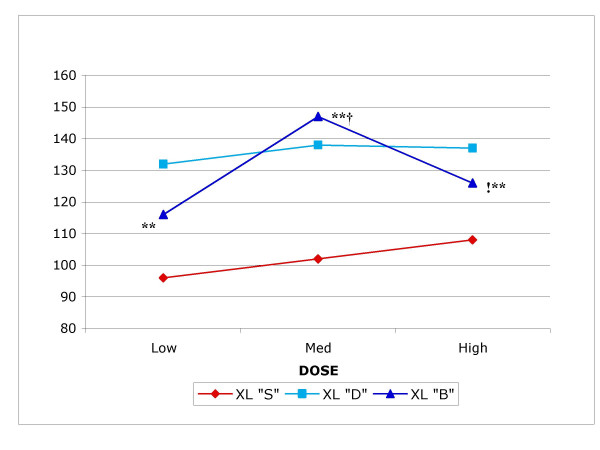
Effect of three different doses of mucomodulator XL "B" (sodium tetraborate, five dogs), XL"D" (high molecular weight dextran, six dogs) and XL "S" (normal saline solution, sham exposure, two dogs) on normalized tracheal mucus velocity in spontaneously breathing anesthetized dogs. **Newman-Keuls: p < 0.01 with respect to control. ! Bonferroni: p < 0.05 (correlated groups). † Dunnett: p < 0.01 (treatments *versus *control).

Every dog had its own initial control of tracheal mucociliary linear velocity measured at the beginning of the experiment (baseline). We express the normalized percent of change in TMV after exposure to mucomodulators compared to that initial control (NTMV%). In general, the NTMV% data indicate a sustained 40% mean increase in tracheal mucociliary clearance rate in the mucomodulator treated dogs after using the three different doses of both experimental compounds, compared to up to 7% increase in sham exposed dogs (Figure 2).

For NTMV% in sham exposed dogs *versus *those exposed to XL "B" and to XL "D", the Newman-Keuls test showed that there was a significant statistical difference when comparing control *vs*. XL "B" medium and high dose, as well as between low vs. medium dose at the p < 0.01 level (Figure 2). The Bonferroni correction indicated a significant difference only when comparing control *vs*. XL "B" high dose at the p < 0.05 level. The Dunnett's procedure (treatment versus control) also indicated a statistical significant difference only with the XL "B" medium dose at the p < 0.01 level.

However, while performing the control measurement of tracheal mucus clearance we observed that a group of five dogs (40%), of either sex, presented an average control TMV of 2.0 mm/min (2 with XL "D", 2 with XL "B" and 1 with XL "S") compared to an average control rate of 4.6 mm/min in the eight (8) remaining experimental animals.

**Figure 3:**
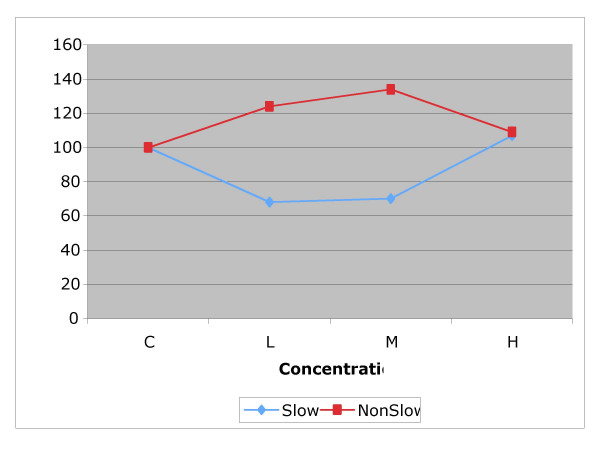
Effect of normal saline solution on tracheal mucus velocity in two spontaneously breathing anesthetized control dogs. Two measurements per dose.

**Figure 4:**
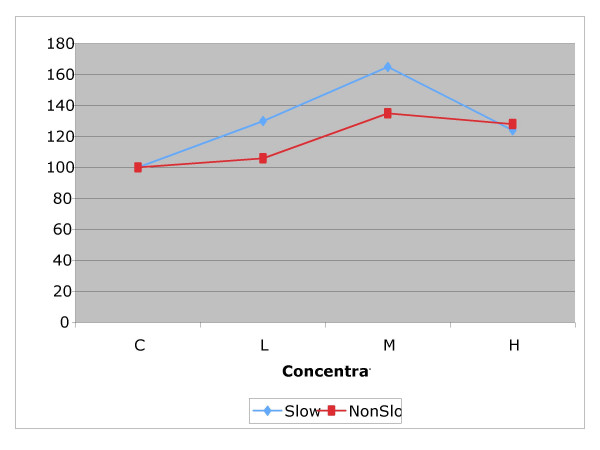
Comparison of the effect of different concentrations of sodium tetraborate (XL "B") on dogs with *slow *versus *non-slow *tracheal mucus velocity. The effect is larger in the *slow *group.

**Figure 5:**
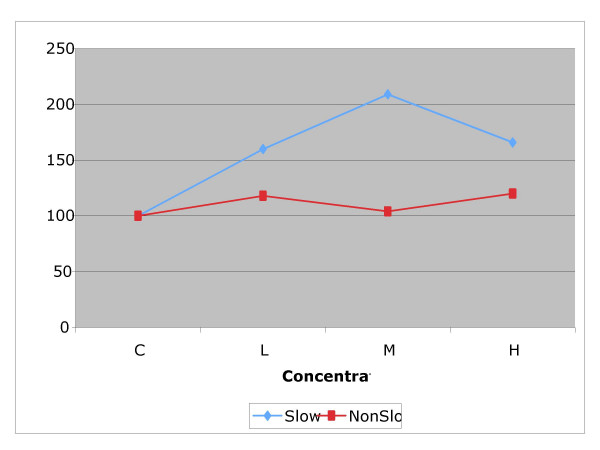
Comparison of the effect of different concentrations of high molecular weight dextran (XL "D") on dogs with slow versus *non-slow *tracheal mucus velocity. The effect is larger in the *slow *group.

Within each experimental drug group, we grouped animals in two separate subgroups: those with *slow *and *non-slow *TMV based on the initial tracheal clearance rate (Figures 3, 4 and 5). Then we carried out a further *t*-test analysis of NTMV% comparing control *versus *dose used as well as between doses within the *slow *and *non-slow *subgroups in each of the three exposed groups: sham, "D" and "B" (Table 1).

**Table 1:** Percentage of Change of TMV Compared with Baseline

	**XL "S"**			**XL "D"**			**XL "B"**		
**Baseline**	100	100	100	100	100	100	100	100	100

**Slow**	-32	-30	+9	+60	+109*	+66^**∞**^	+30	+65	+28*
**Non-Slow**	+24	+34	+9	+18	+4	+20	+6	+35^**§**^	+24

* = p < 0.05, compared with Baseline

^**§ **^= p < 0.01, compared L *vs*. M doses XL "D", **∞ **= p < 0.06, compared with medium dose XL "D"

## Discussion

The focus of this study is on the physiological function of the mucociliary clearance apparatus with and without exposure to mucomodulators in both the *ex-vivo *epithelial model and the *in-vivo *large mammal model.

Major findings in this study include: a) The two epithelial models used in this study, the *ex-vivo *frog palate and the *in-vivo *mammalian model, appear to be appropriate and complement each other to study the effects that our mucomodulators exert on the mucociliary clearance defence mechanism; b) the mucin macromolecules of the herein used *in-vivo *mammalian model (dog) showed selective crosslinking behavior after exposure to mucomodulators as it was previously shown in mucus simulants: increased elasticity, expressed by increased thread formation capacity and is more easily clearable from intact airways; c) the physiological function of the mucociliary apparatus was not negatively affected in either of the two epithelial models; d) modulated airway mucus is better cleared from an intact airway system and normally functioning respiratory system after exposure to mucomodulators; e) data presented in this study allow us to assure that we have complied with the fundamental requirement criteria established in the initial phase of developing the concept of mucomodulation: Can we modulate the physical characteristics of the respiratory secretions to reduce aerosolization without affecting normal mucociliary clearance function, or even better improving it? [9].

In this study we combined the use of two very different ciliated epithelial models to have a better assessment of the effect that crosslinking agents have on clearance of mucus by interaction with cilia. Several observers had raised questions previously about the adequacy of the frog palate as a model to assess airway mucus clearance, since in the first place it belongs to a non-mammalian species, in addition that it is non-respiratory. However, this ciliated epithelium has a well developed mucus blanket that works in coordination with cilia similar to that observed in other mammals including humans. This epithelial model also permits us to assess mucus clearance by cilia interaction only without the airflow factor [15].

The *in-vivo *mammalian model, using anesthetized dogs, allowed us to assess the effects that our mucomodulators exert on the mucociliary clearance defence mechanism in an intact airway system and normally functioning respiratory system. The intact airway system in our dog model permitted us to also assess the potential contribution of other factors in clearing a modulated mucus, particularly airflow during normal spontaneous breathing.

Data obtained from the dogs showed that inhalation of three different doses of mucomodulators did not adversely affect the cardio-respiratory pattern even after the different procedures involved and several hours that lasted every experiment. The data support the observation that cilia were not functionally affected after exposure to mucomodulators.

After aerosolizing the mucomodulators and while collecting the samples of mucus, we observed an increase in thread formation capacity of the mucus even when using low concentration doses. Since we did not have a Filancemeter on site, we anchored the mucus in a fang of the dog and used a wooden pick or a needle to pull the mucus and observed it stretching and forming long threads before rupture. With no mucomodulators, the mucus threads ruptured after stretching up to 5 to 8 mm, while it ruptured after stretching around 30 to 50 mm following exposure to mucomodulators.

During and after aerosolizing the mucomodulators and while collecting the samples of mucus, we examined the airways of the experimental dogs, as far down in the airway tree as the bronchoscope allowed access, to visually assess bronchial patency and possible obstruction of the airways caused by the modulated mucus. We observed the airways in all experimental animals free of obstruction throughout the study period. All dogs, either exposed to placebo or mucomodulators, maintained a normal respiratory rhythm and frequency throughout the experiment that might suggest they were free of respiratory distress. However more studies on this topic are required to arrive to a more definite conclusion.

After showing in the mammalian model that instead of deterioration of the mucociliary clearance mechanism there was a clear improvement in tracheal mucus clearance, we carried out an additional trial to assess the effect of those mucomodulators on cough and aerosol generation pattern in the same dog model. After 20 minutes of nebulization of capsaicin solution, the dog did not cough or sneeze at all. We aborted the experiment after that time and after the researchers were constantly coughing. We still cannot find the reasons why capsaicin did not make the dog cough. Veterinarians consulted about this event found no answer either.

The modulated "thicker" mucus neither plugged the airways of the experimental (mammalian) subjects, as far down in the airways tree as the bronchoscope allowed us to assess, or was the tracheal mucus clearance rate negatively affected. On the contrary and unexpectedly, tracheal mucus velocity systematically increased even with the lowest dose of either mucomodulator administered.

The observations addressed in the previous paragraph may help to respond to the greatest concern expressed by some researchers and clinicians when they first learn about mucomodulators, "*that crosslinking the mucus layer in this way, will make mucus 'thicker' and might obstruct the airways in exposed subjects*". We have started to build the evidence that the proposed drugs that increase the concentration of crosslinking sites in the mucin glycoprotein gel network do not gum up airway secretions, do not plug the airways and have no adverse effect on the clearance of airway mucus in a normally functioning respiratory system of a mammalian model. However, the mucomodulators we tested in this study might still have some effects in areas that were not visible or measurable through the bronchoscope, such as in smaller airways or alveolar tissue. More studies are required before reaching a definite conclusion to rule out side-effects of the mucomodulators on respiratory secretions of smaller airways and alveolar tissue or on gas exchange.

Since mucus clearance in the airways is a function of the intimate interaction of the anatomophysiological bicellular unit, ciliated cells and mucus producing cells, this study has allowed us to determine that no functional disruption of the normal clearance of mucus occur when we selectively alter the elastic property of mucus.

The *ex-vivo *frog palate model is an excellent model for assessing the effect of the mucomodulators on the mucus-cilia interaction undisturbed by internal or external factors like airflow. The *in-vivo *mammalian model is an excellent model for assessing in an integral manner the effect of the mucomodulators on the mucus interacting with cilia as well as with several other respiratory components like airflow during normal breathing.

Selective modulation of the elastic properties of mucus in our mammalian model indicates that the modified macromolecule of mucin is engineered to work in an exquisite manner with the low speed airflow during normal breathing in a normally functioning respiratory system.

Several years ago we had the opportunity to analyze a sample of tracheobronchial mucus of a patient diagnosed with primary ciliary dyskinesia and the mucus showed to have predominantly more elastic properties. Individuals with this genetic syndrome depend completely on airflow interaction for airway mucus clearance since they have a totally dysfunctional cilia apparatus, i.e. immotile cilia. This finding guided us in our approach to develop the mucomodulators.

An additional mechanism to explain the enhanced mucus clearance observed in our mammalian model could be that inhaled mucomodulators stimulate cells producing surfactant factor. The possible presence of a surfactant factor both within and between the sol and gel phase of the mucus may play a role in facilitating mucus clearability or cough clearability, by allowing the mucus layer to slide easily and more effectively on top of the cilia. This may explain the observation of mucus sliding in an easier manner from the endotracheal tube surface.

There may occur a combination of these hypotheses, as well as other possible new mechanisms, such as the increased osmolarity composition of the aerosolized solutions enhancing airway secretions hydration. However, given the fact that tracheal mucus velocity peaked with the intermediate dose of XL "B" (equivalent of 1.0% NaCl), and not with the highest dose (equivalent of 1.8% NaCl), the contribution of hyperosmolarity is not likely the major explanation for the stimulation of TMV. We anticipate that further work is needed to clearly define the mechanisms involved in the enhanced clearance of airway secretions when exposed to our mucomodulators.

Both mucomodulators used in this study exhibited an effect in modulating the mucus macromolecule of the airways of the dogs by increasing elasticity. We may rightly assume from the latter that poorly soluble mucin complex or more cohesive mucus have been formed that will be more resistant to break up during high speed airflow interaction, hence expelling less particles as aerosol.

For centuries people around the world from different non-related cultures have been extensively searching plants, minerals and animal products to best improve functions and defence mechanisms for pulmonary disorders involving abnormal mucus and hypersecretion. In this study we have successfully used a natural product: high molecular weight dextran, a bacterial biopolymer produced by the *Leuconostoc mesenteroides *that showed promising properties to enhance airway hygiene and airborne infectious diseases.

Results from this study indicate that the critical measures of success, namely to maintain airway clearance has been proved in both *ex-vivo *as well as *in-vivo *epithelial models. The degree of improvement in tracheal mucociliary clearance using our mucomodulators is far better than that achieved by using current approaches in the same animal model. Our mucomodulators can be considered as an enhanced new generation of compounds able to improve airway hygiene and respiratory host defence.

Data in this study complement with our data previously published to support the concept that our mucomodulators can achieve a dual goal of reducing aerosolization without impairing lung clearance, since the two most important clearance mechanisms of the respiratory system remain functional. Mucomodulators have no reported antitussive action hence no cough mechanism is affected.

## Conclusion

We have acquired preliminary evidence that mucomodulators that increase cohesive interactions between mucin macromolecules make mucus more easily clearable, most likely by airflow-dependent mechanisms. Increasing the cohesiveness in this manner might help to reduce fine aerosol formation during coughing or sneezing – a desirable advantage in helping to control the spread of airborne infections.

Mammalian subjects in this study showed a very different tracheal mucus velocity from other studies using a similar model. We do not have an explanation for this matter. Both mucomodulators enhanced tracheal mucus velocity in all those exposed. However, tracheal mucus velocity was best enhanced in those with slower mucus clearance. This finding might be of particular use for those individuals who have impaired mucociliary clearance.

We anticipate other potential benefits that relate to enhancing mucus clearance, a major consideration in conditions of impaired airway secretion management i.e. patients suffering from COPD, spinal cord injury, neuromuscular disease or those in intensive care units and/or those who require assisted mechanical ventilation.

These findings might assist us in enhancing the knowledge of the changes in the mucociliary apparatus that occur after exposure to mucomodulator products, with the goal to understand how these changes relate to an improved and innovative management of chronic airway pathologies in humans.

## Competing interests

The authors declare that they have no competing interests.

## Authors' contributions

MK and GZ developed the concept of Mucomodulation, designed the study, interpreted the data and drafted the manuscript.

MR, a Virologist graduated from Karolinska University, Sweden, has contributed with valuable intellectual concepts in his field of expertise for GZ to strengthen the study. MR was involved in the data acquisition during all the experiments and played a key role to supervise and guarantee the quality of data acquisition.

JCV (Resident Year 1), Mauricio Alonso (Resident Year 2), HA (Resident Year 3) and DV (Chief of Residents) all enrolled in the Neumology (Respiratory Medicine) Residency Program, in the Hospital Nacional "Dr. Jose Antonio Saldaña" Neumologia y Medicina Familiar. They all were trained and supervised by GZ in the research methodology and procedures in large mammals (dogs), including animal preparation, airway intubation, bronchoscopic procedures, measurements of TMV, mucus collection, mucomodulator administration and open chest surgery for tracheobronchial surgical resection for pathological studies. They all performed all these procedures.

GB, a Registered Nurse, organized and prepared the research site, catheterized front leg vein of the dog and administered anesthesia as required to keep the animal lightly anesthetized, set up and procured research supplies, and assisted researchers to maintain control of time and to run the experiments smoothly. Due to the various research procedures and time requirements, her contributions were very valuable in successfully running the experiments.

All authors have read and approved the final manuscript.

## Pre-publication history

The pre-publication history for this paper can be accessed here:



## References

[B1] Silberberg A, Meyer FA, Chantler EN, Elder JB, Ebstein M (1982). Structure and function of mucus. Mucus in Health and Disease II.

[B2] Basbaum CB, Finkbeiner WE, Massaro D (1989). Mucus-producing cells of the airways. Lung Cell Biology.

[B3] King M, Rubin BK, Takishima T, Shimura S (1994). Rheology of airway mucus: Relationship with clearance function. Airway Secretion: Physiological Bases for the Control of Mucus Hypersecretion (Lung Biology in Health and Disease Series).

[B4] Zayas JG, Rubin BK, York E, Lien D, King M (1999). Bronchial mucus properties in lung cancer. Relationship with site of lesion. Can Respir J.

[B5] Lopez-Vidriero MT (1987). Biochemical basis of physical properties of respiratory tract secretions. Eur J Respir Dis.

[B6] King M, Rubin BK (2002). Pharmacological approaches to discovery and development of new mucolytic agents. Advanced Drug Delivery Reviews.

[B7] King M, Braga PC, Allegra L (1988). Magnetic microrheometer. Methods in Bronchial Mucology.

[B8] King M (1987). Role of mucus viscoelasticity in cough clearance. Biorheology.

[B9] Zayas G, Dimitry J, Zayas A, O'Brien D, King M (2005). A new paradigm in respiratory hygiene: increasing the cohesivity of airway secretions to improve cough interaction and reduce aerosol dispersion. BMC Pulm Med.

[B10] King M, Festa E, Baum G (1998). The evolution of the frog palate model for mucociliary clearance. Cilia, Mucus and Mucociliary Interactions.

[B11] Rubin BK, Ramirez O, King M (1990). Mucus-depleted frog palate as a model for the study of mucociliary clearance. J Appl Physiol.

[B12] Jeanneret-Grosjean A, King M, Michoud MC, Lioté H, Amyot R (1988). Sampling technique and rheology of human bronchial mucus. Am Rev Respir Dis.

[B13] Rubin BK, Ramirez O, Zayas JG, Finegan B, King M (1990). Collection and analysis of respiratory mucus from individuals without lung disease. Am Rev Respir Dis.

[B14] King M, Zahm JM, Pierrot D, Vaquez-Girod S, Puchelle E (1989). The role of mucus gel viscosity, spinnability, and adhesive properties in clearance by simulated cough. Biorheology.

[B15] Zayas G, O'Brien DW, Tai S, Ding J, Lim L, King M (2004). Adaptation of an Amphibian Mucociliary Clearance Model to Evaluate Early Effects of Tobacco Smoke Exposure. Respir Res.

